# Carbon-Based Electrochemical (Bio)sensors for the Detection of Carbendazim: A Review

**DOI:** 10.3390/mi14091752

**Published:** 2023-09-07

**Authors:** Constanza J. Venegas, Soledad Bollo, Paulina Sierra-Rosales

**Affiliations:** 1 Programa Institucional de Fomento a la Investigación, Desarrollo e Innovación, Universidad Tecnológica Metropolitana, Ignacio Valdivieso 2409, San Joaquín, Santiago 8940577, Chile; 2 Centro de Investigación de Procesos Redox (CiPRex), Universidad de Chile, Sergio Livingstone Polhammer 1007, Independencia, Santiago 8380492, Chile; 3 Advanced Center for Chronic Diseases (ACCDiS), Universidad de Chile, Sergio Livingstone Polhammer 1007, Independencia, Santiago 8380492, Chile

**Keywords:** carbendazim, carbon nanotubes, graphene, carbon nitride, electrochemical biosensors

## Abstract

Carbendazim, a fungicide widely used in agriculture, has been classified as a hazardous chemical by the World Health Organization due to its environmental persistence. It is prohibited in several countries; therefore, detecting it in food and environmental samples is highly necessary. A reliable, rapid, and low-cost method uses electrochemical sensors and biosensors, especially those modified with carbon-based materials with good analytical performance. In this review, we summarize the use of carbon-based electrochemical (bio)sensors for detecting carbendazim in environmental and food matrixes, with a particular interest in the role of carbon materials. Focus on publications between 2018 and 2023 that have been describing the use of carbon nanotubes, carbon nitride, graphene, and its derivatives, and carbon-based materials as modifiers, emphasizing the analytical performance obtained, such as linear range, detection limit, selectivity, and the matrix where the detection was applied.

## 1. Introduction

**Figure 1:**
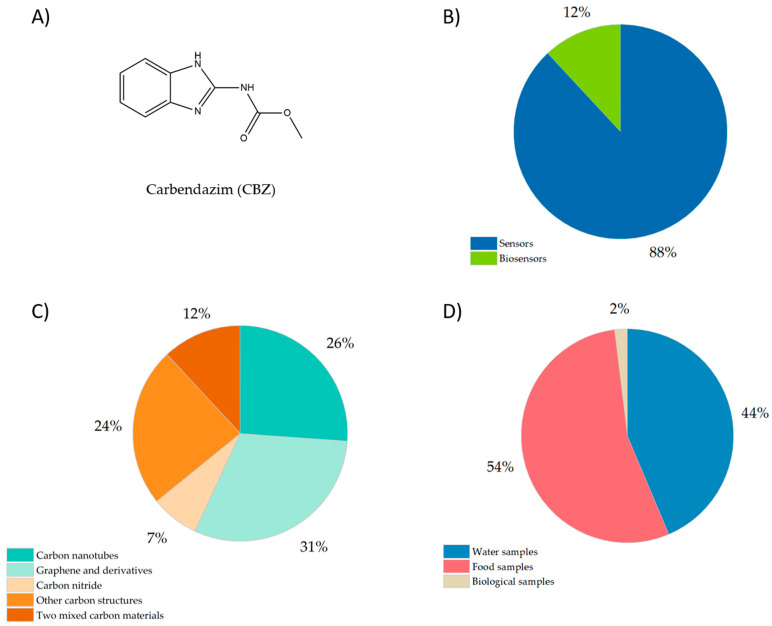
(**A**) Chemical structure of carbendazim (CBZ); (**B**) pie chart showing the relative distribution of carbon-based electrochemical (bio)sensors for the detection of CBZ; (**C**) pie chart showing the relative distribution of carbon nanomaterials used in electrochemical (bio)sensors for the detection of CBZ, and (**D**) pie chart with the relative distribution of samples matrixes where CBZ has been detected. (Articles published between 2018 and 2023).

One widely used pesticide is carbendazim (CBZ), 2-benzimidazole methylcarbamate (Figure 1A), a fungicide that is highly stable with a low degradation rate, making it persistent in soil, water, and food. That is why the Environment Protection Agency has declared the maximum residue level (MRL) of CBZ as 0.5 mg/kg in agricultural products and between 100 and 700 ppb in citrus fruits; meanwhile, some countries have banned its usage [1]. 

Conventional analytical methods used for CBZ quantification are based on chromatographic techniques, which provide sensitive and selective detection. However, these techniques require highly skilled technicians; they are unsuitable for screening analysis and demand laborious pretreatment of samples to extract the compound analyzed from the matrix [2,3,4]. On the other hand, electrochemical (bio)sensors have become an interesting platform due to their fast response, high sensitivity, excellent precision and accuracy, specificity, low cost, easy handling, multiple capabilities, and field applicability [5,6,7]. One of the factors that has most contributed to the improvement in the performance of these electrochemical (bio)sensors has been the incorporation of nanomaterials on the electrode surface.

Nanomaterials have at least one dimension of their size between 1 and 100 nm. They can form nanoparticles, nanorods, nanofibers, nanotubes, etc. The most commonly used nanomaterials include carbon nanotubes (CNTs), graphene, metal nanoparticles (MNPs), metal oxide nanoparticles (MONPs), and quantum dots (QDs), among others. In electrochemistry, these nanomaterials provide advantages such as increasing electroactive areas and decreasing overpotentials [8,9,10]. All these characteristics make these nanomaterials fundamental elements in electroanalysis, nanoscience, and nanotechnology.

As shown in Figure 1B, according to the literature (2018–2023), of the total articles published on the electrochemical determination of CBZ using carbon nanomaterials, 88% correspond to sensors and 12% to biosensing platforms. Figure 1C shows more detailed information about the type of carbon nanomaterials used, where carbon nanotubes (CNT) and graphene derivatives represent 57% of the total articles, 24% use other carbon structures such as carbon spherical shells (CSS), carbon nanofibers (CNFs), N-doped carbon (NC), porous carbon derivatives, carbon nanospheres (CNS), and carbon nanohorns (CNH)s, 12% combine two carbon materials searching for a synergistic effect that improves the sensor’s response, and 7% use carbon nitride as modifier. Finally, Figure 1D shows the matrixes where the electrochemical (bio)sensors were applied to determine CBZ, observing that the detection of CBZ has been mainly focused on water and food samples.

To date, three reviews have been reported for the detection of CBZ. One, in 2020, made a compilation of different chromatographic methods for detecting and quantifying CBZ in food, highlighting the matrices, implementing extraction steps, and chromatographic techniques [11]. In 2021, a second review of nanomaterials included carbon nanotubes, graphene hybrids, MXenes, and metal nanoparticles based on non-enzymatic and operational electrochemical sensors for CBZ [12]. Finally, in 2023, advances in nanomaterial-based optical sensors, such as colorimetric, fluorescence, and surface-enhanced Raman scattering, will be made for the monitoring of benzimidazole fungicide (including carbendazim) residue in food and water samples [13]. In the following sections, we provide a comprehensive review focused on articles published since 2018 for carbon-based electrochemical sensors and biosensors for CBZ, with special emphasis on sensor construction and reporting the lowest LOD values.

## 2. Methodology

The following review was carried out following the guidelines proposed by the PRISMA statement for reporting systematic reviews and meta-analyses [14]. The inclusion and exclusion criteria were: The search was limited to articles in English that included the study keywords. The investigation was focused on the following keywords: “carbendazim”, “electrochemical detection”, and “carbon-based materials”. The search was extended to recent carbon-based (bio) sensor articles. The query included “carbendazim AND electrochemical detection AND carbon-based material”, “carbendazim AND electrochemical detection AND carbon-based (bio)sensor”, “carbendazim AND sensor”, “carbendazim AND biosensor”, “carbendazim AND carbon-based electrode”. Only articles with original research published between 2018 and May 2023 using at least one carbon-based material for the electrochemical detection of carbendazim were considered. Investigations detecting carbendazim by methods other than electrochemical and using materials other than carbon-based materials were excluded. Search strategy: ScienceDirect, Scopus, and Google Scholar were used as electronic databases. Forty-two articles that met the search criteria mentioned above were selected. Of those, 37 corresponded to electrochemical sensors for CBZ detection, classified according to the carbon-based material used, and five were related to electrochemical biosensors for CBZ detection.

## 3. Electrochemical Sensor for CBZ Detection

### 3.1. Sensors Based on Carbon Nanotubes

Carbon nanotubes (CNTs) are sp^2^ carbon structures made up of graphene sheets arranged as molecular cylinders. They can be mainly found as single-walled carbon nanotubes (SWCNT), double-walled carbon nanotubes (DWCNT), and multi-walled carbon nanotubes (MWCNTs). These nanomaterials have high chemical and thermal stability, low capacitance, increased peak currents, and decreased overpotentials, which improve the sensitivity of the sensors [12,15,16,17]. Among CNTs, as shown in Table 1, the electrochemical sensors developed for CBZ detection are based on MWCNT and were applied to water and food samples. 

**Figure 2:**
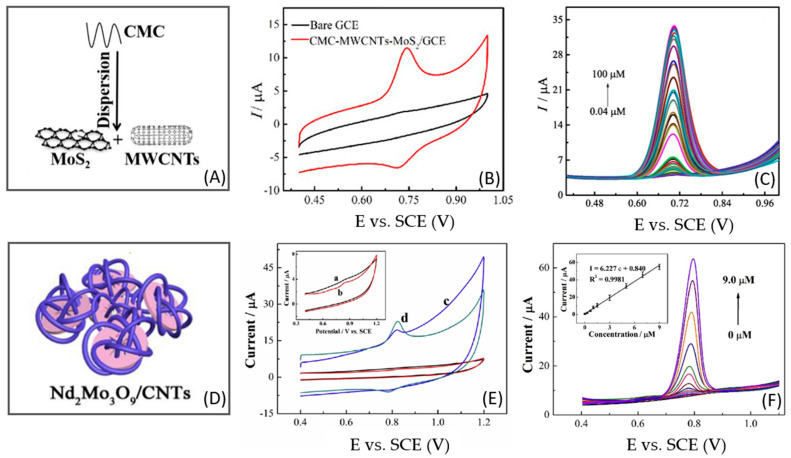
(**A**) Scheme of nanohybrid of MoS_2_/MWCNTs; (**B**) cyclic voltammogram of 10 μM CBZ at bare GCE and MoS_2_/CMC-MWCNT/GCE; (**C**) DPV of CBZ at different concentrations in 0.1 M PBS at MoS_2_/CMC-MWCNT/GCE; (**D**) Scheme of nanohybrid of Nd_2_Mo_3_O_9_/MWCNT; (**E**) cyclic voltammogram of 1.5 µM CBZ at (a) bare GCE, (b) Nd_2_Mo_3_O_9_/GCE, (c) MWCNT/GCE, and (d) Nd_2_Mo_3_O_9_/MWCNT/GCE; (**F**) DPV curves of different concentrations of CBZ in 0.1 M PBS (pH 7.0) at Nd_2_Mo_3_O_9_/MWCNT/GCE. Reproduced with permission from Refs. [18,20].

Zhu et al. [18] developed an electrochemical biomimetic sensor coupled with machine learning using a nanohybrid of MoS_2_/MWCNTs. The aqueous dispersion of the nanohybrid was prepared with sodium carboxymethyl cellulose (CMC), which conferred stability to the film and did not affect the electron transfer. The dispersion was used to modify a glassy carbon electrode (GCE), and then the sensor was used to detect CBZ in 0.1 M PBS (pH 7.0). As shown in Figure 2A–C, the nanohybrid MoS_2_/CMC-MWCNTs significantly increase the surface area of the electrode, whereas CBZ showed a reversible electrochemical process. By differential pulse voltammetry (DPV), the MoS_2_/CMC-MWCNT/GCE could detect CBZ in a wide linear range of 0.04–100 μM, with a LOD of 7.4 nM and an RSD of 1.12%, showing good reproducibility. The sensor presented good selectivity when CBZ was detected in the presence of vitamin C, vitamin B2, imidacloprid, glyphosate, endosulfan, buprofezin, fructose, sucrose, L-arginine, and L-serine. Finally, to evaluate the application of the sensor, CBZ was detected in tea and rice with recoveries between 89.2 and 105.6%, demonstrating reasonable practicability.

In the case of Malode et al. [19], the authors obtained a nanocomposite based on multi-walled carbon nanotubes and calcium-doped zinc oxide to modify a carbon paste electrode (MWCNT/Ca-ZnO-CPE). The sensor was used to determine CBZ in 0.2 M PBS (pH 7.0), showing an electrocatalytic behavior increasing five times the electrode’s current compared to the bare CPE. For the analytical application, the electrooxidation of CBZ at MWCNT/Ca-ZnO-CPE was followed by square wave voltammetry (SWV), obtaining a linear range of 0.01–0.45 μM and a LOD of 4.7 nM. Although the authors did not evaluate the effect of interfering species, they applied the sensor to estimate CBZ in soil and water samples. The samples were spiked with a known amount of CBZ, obtaining recoveries between 81.0 and 94.3% for soil samples and 92.5 and 96.2% for water samples.

Finally, one of the lowest LOD values obtained for CBZ using electrochemical sensors based on carbon nanotubes was reached with a glassy carbon electrode modified with a flake-like neodymium molybdate wrapped with MWCNT (Nd_2_Mo_3_O_9_/MWCNT/GCE) [20] (Figure 2D–F). 

The detection of CBZ was conducted with the bare GCE and modified GCEs, where the oxidation peak of CBZ increased 3.2 times at Nd_2_Mo_3_O_9_/MWCNT/GCE compared to Nd_2_Mo_3_O_9_/GCE. This result verifies a synergistic effect between Nd_2_Mo_3_O_9_ and MWCNT, where Nd_2_Mo_3_O_9_ increases the active sites and MWCNT enhances the electron transfer capability of the composite. Under the optimized experimental conditions, the detection of CBZ was conducted in 0.1 M PBS (pH 7.0) using DPV at Nd_2_Mo_3_O_9_/MWCNT/GCE. A linear range of ${5\times 10}^{-5\;}–9.0$ µM and a LOD of 0.016 nM were obtained. The sensor proved to be reproducible (RSD 4.35%), stable, and selective when CBZ was mixed with $K^{+},\;{Na}^{+},\;{NH}_{4}^{+},\;{Cu}^{2+},\;{{Cd}^{2+},\;Al}^{3+},\;{Cl}^{-},\;{CO}_{3}^{2-},\;{PO}_{4}^{3-},\;{SO}_{4}^{2-}$ methyl parathion, fenitrothion, malathion, dichlorophenol, benomyl, thiabendazole, thiophanate, thiophanate-methyl, fuberidazole, glucose, ascorbic acid, Vitamin B, C, E, dopamine, and serine. The practical application of the sensor was the detection of CBZ in water samples, obtaining 96.7–102.0% of recoveries with an RSD of 3.68%.

### 3.2. Sensors Based on Graphene and Its Derivatives

Graphene and graphene-derivative materials are based on a sheet of sp^2^-hybridized carbon atoms in a honeycomb structure. Similar to CNTs, they present valuable characteristics such as a high electron-transport window, a large surface area, a tunable electronic band, high mechanical strength, and good thermal conductivity [12,21,22]. Among the most commonly used graphene derivatives, we can find graphene oxide (GO), reduced graphene oxide (rGO), graphene nanosheets (GS), and electrochemically reduced graphene oxide (ERGO). For the electrochemical detection of CBZ, the lowest LOD values were reached with GCE modified with GS and ERGO and were applied to water and food samples.

Wei et al. [21] prepared graphene nanosheets (GS) via ultrasonic exfoliation of graphite in the presence of N-methyl-2-pyrrolidone (NMP) and modified a glassy carbon electrode with GS suspension (GS/GCE) to follow the oxidation of CBZ. The GS/GCE was used to determine the oxidation behavior of 20 mg L^−1^ CBZ in 0.1 M PBS (pH 5.7). By DPV, a single oxidation response was obtained with GS/GCE; meanwhile, bare GCE shows no peak related to CBZ. The GS/GCE showed smaller charge transfer resistance by EIS, verifying the material’s good electron transfer ability. Under the optimized conditions (5.0 µL GS suspension and accumulation at 0.50 V for 5 min), a linear range was found between 0.005 and 1.57 µM with an LOD of 0.78 nM (0.15 µg L^−1^). Additionally, the sensor was used in the presence of Cu^2+^, Zn^2+^, Cd^2+^, Pb^2+^, Hg^2+^, hexachlorobenzene, folpet, triclosan, picloram, hydroquinone, phenol, bisphenol A, tetrabromobisphenol A, parachlorophenol, p-aminophenol, parathion, and Dursban, obtaining a peak current change of less than 5%. Finally, the GS/GCE detected CBZ in groundwater, soil, and cucumber samples. Although CBZ was not detected in these samples, different concentrations of CBZ were spiked, obtaining a recovery range between 96.2 and 101.5%, suggesting good accuracy.

**Figure 3:**
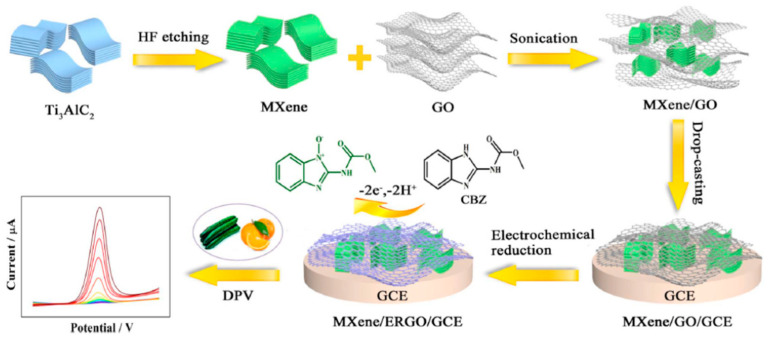
Scheme of sensor based on graphene Ti_3_C_2_T_x_/ERGO/GCE. Reproduced with permission from reference [23].

Xie et al. [23] synthesized a transition-metal carbide (MXene) with ERGO to obtain a conductive composite and evaluate its application for the electrochemical detection of CBZ. As shown in Figure 3, the composite was synthesized by mixing Ti_3_C_2_T_x_ (MXene) and GO by sonication, using GO as spacers between MXenes. The composite was dropped on the electrode, and then GO was electrochemically reduced to obtain MXene/ERGO. This composite presents enhanced electronic conductivity, improving the sensor’s response. The voltammetric behavior of CBZ in 0.1 M PBS (pH 7.0) was evaluated by CV using bare GCE, MXene/GCE, ERGO/GCE, and MXene/ERGO/GCE, where the last electrode showed the larger currents due to the synergistic effect between ERGO and MXenes. By DPV and under optimized conditions (5.0 µL MXene/ERGO suspension and accumulation at 0.30 V for 180 s), a linear range was obtained between 0.002 and 10 µM, with a LOD value of 0.67 nM. The selectivity of the sensor was evaluated in the presence of $K^{+},\;{Na}^{+},\;{Mg}^{2+},\;{Cr}^{3+},\;{Al}^{3+},\;{NO}_{3}^{-},\;{CO}_{3}^{2-},\;{PO}_{4}^{3-},$ malathion, methyl paration, imidacloprid, trichlorfon, and propham, where none of them impact the detection of CBZ. The applicability of the sensor was evaluated by the standard addition of CBZ to cucumber and orange juice samples, with recoveries ranging from 97.6% to 103.8%.

Elshafey et al. [24] developed a sensor based on cobalt oxide/ERGO using imprinted polypyrrole (PPy) film. First, the GCE was modified with ERGO and then with CoOx by electrodeposition. After that step, PPy was electrosynthesized with CBZ as a template. CV and EIS confirmed the synergistic properties of the CoOx/ERGO mix in the presence of a ${{\left[Fe(CN\right)}_{6}]}^{3-/4-}$, where the sensor showed a significant conductivity improvement and facilitated the redox mediator’s electron transfer process. Concerning CBZ, the electrocatalytic behavior of the electrodes was evaluated by CV and DPV in the presence of 50 µM CBZ in 0.04 M Britton-Robinson buffer (pH 2.0). Compared to GCE, the sensor CoOx/ERGO/GCE showed an increase in current of 6.5 folds. Considering that CoOx is a semiconductor, when anchored to ERGO, the surface area is enhanced, which improves its catalytic activity. In a second stage, the authors obtain a PPy MIP-based sensor by electropolymerizing pyrrole in the presence of CBZ on the CoOx/ERGO/GCE, obtaining a final electrode of PPy-CoOx/ERGO/GCE. The MIP-based sensor proved sensitive to CBZ, obtaining peak currents of about 2.3 fold compared to the sensor without the MIP. At optimal conditions, a calibration plot of CBZ was conducted with a linear range of 0.01 nM to 10 µM and a LOD of 0.01 nM. In this sensor, ERGO gives high conductivity, CoOx improves the specific surface area, and both provide higher loading of MIP cavities for CBZ. The selectivity of the MIP-based sensor was proven to detect CBZ in the presence of thiophanate-methyl, metalaxyl, alachlor, dimethoate, chlorpyrifos, and profenofos. An insignificant change in current was recorded, indicating the good selectivity of the sensor. The CBZ-MIP sensor was applied to detect CBZ in tomato and apple samples, obtaining recoveries from 92.2 to 101.0%, demonstrating the lack of matrix effects.

### 3.3. Sensors Based on Carbon Nitride

Graphitic carbon nitride (g-C_3_N_4_) was synthesized for the first time in 1834 [25]. It is a semiconductor allotrope of the carbon nitrides family composed of tertiary ammonia crosslinked triazine with a planar structure related to graphite or graphene, and the p-conjugated graphitic planes are formed by C and N atoms with hybridization sp^2^ [26,27]. It has a structure with highly stable mechanical and physicochemical properties and stands out in applications in energy and catalysis, mainly in research in photocatalysis, including the degradation processes of organic compounds, CO_2_ reduction, and water splitting [28,29]. Other applications, such as ionic actuators, supercapacitors, lithium-ion batteries, drug delivery, and electrochemical sensors, can also be found [29,30]. 

Due to the properties of g-C_3_N_4_, they have allowed its use in various fields of application. However, it presents disadvantages, such as a reduced surface area due to the weak interaction in the conjugated π-π structure [31]. Among the strategies used to increase this material’s size and catalytic performance, the doping and substitution of metals, non-metals, and heteroatoms stand out through the thermal process with NaBH_4_ under an inert atmosphere [28,32]. In electrochemical sensing, the high-sensitivity detection of a wide variety of analytes such as H_2_O_2_ [33], nitroaromatic compounds [34], pharmaceutical samples [35], and agricultural pollutants such as carbendazim has been reported.

**Figure 4:**
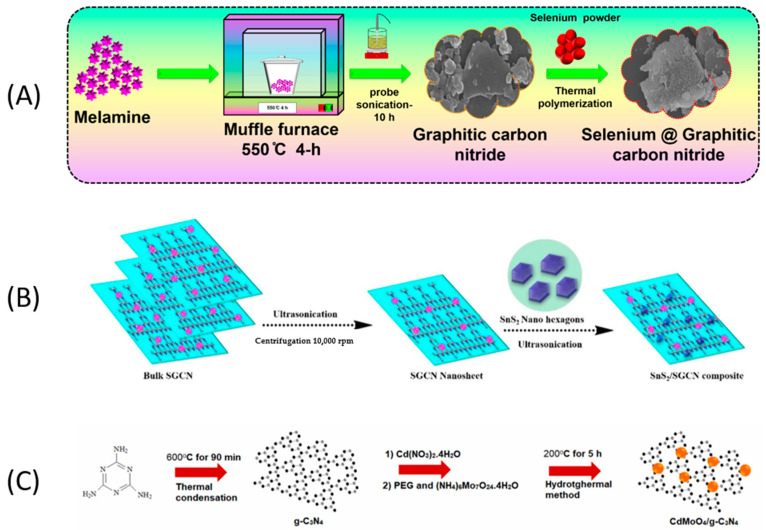
Schematic representation of the preparation of g-C_3_N_4_ composites. (**A**) Se-g-C_3_N_4_; (**B**) SnS_2_/g-C_3_N_4_; and (**C**) CdMoO_4_/g-C_3_N_4_. Reproduced with permission from ref. [1,36,37].

Nataraj et al. [1] improved the efficiency of g-C_3_N_4_ by substituting selenium (Se) into the triazine ring as Se-g-C_3_N_4_, based on the redox activity of Se that can facilitate electron capture in an electrochemical process. The synthesis of g-C_3_N_4_ was performed via a thermal polymerization method of melamine at 550 °C for 4 h, followed by an exfoliation step assisted via probe-sonication for 10 h (Figure 4A). Finally, the sample was collected and dried at 50 °C for 12 h in an oven. The substitution of Se in the g-C_3_N_4_ structure was conducted using a mixture of selenium powder (SeO_2_) and g-C_3_N_4_ heated at 550 °C for 4 h as a thermal polymerization method. The samples were collected and dried in an oven at 50 °C overnight. The electrodes were fabricated using a screen-printed carbon electrode (SPCE) modified by drop casting. The surface area of the electrodes was evaluated in the presence of ${\left[Fe(CN\right)}_{6}{]}^{3-/4-}$, where the effective active surface area was increased from 0.078 ${cm}^{-2}$(SPCE) to 0.114 ${cm}^{-2}$ at g-C_3_N_4_/SPCE, and 0.125 ${cm}^{-2}$ at Se-g-C_3_N_4_/SPCE. Under optimized conditions, the CBZ detection was performed by DPV with a LOD of 6.0 nM and a linear range of 0.099–346.9 µM. They studied the sensor’s response against many interferents (4-aminophenol, chloramphenicol, diphenylamine, glucose, hydroquinone, fenitrothion, potassium ions, melatonin, sodium ions, bisphenol-A, methyl parathion, carbofuran, and mercury nitrate), demonstrating the high selectivity of the proposed sensor. Finally, the authors evaluated the detection of CBZ in two enriched samples, orange juice, and vegetable extract water, with a recovery greater than 98%. 

Shanmugam et al. [36] prepared a composite incorporating nanohexagon-shaped tin sulfide (SnS_2_) on sulfur-doped g-C_3_N_4_ (SGCN) (Figure 4B), enhancing the defective sites and its active surface area and diminishing the bandgap, which improved the electrochemical performance. SnS_2_ was obtained hydrothermally, and g-C_3_N_4_ was synthesized from melamine and thiourea heated at 550 °C for 4 h in an air atmosphere. Both nanomaterials were dispersed in water, and the composite suspension was ultrasonicated, centrifuged, and washed. The composite was used to modify SPCEs by drop casting with an aqueous dispersion of SnS_2_/g-C_3_N_4_. The bare electrode showed the oxidation of CBZ at 0.82 V, which decreased to 0.65 V when the electrode was modified with SnS_2_/g-C_3_N_4_. The authors indicate that the defective sites of the composite enhance the CBZ absorption on the electrode, improving the oxidation reaction efficiency. The determination of CBZ was performed by DPV in PBS (pH 7.0), obtaining two consecutive linear ranges (0.02–0.9 μM) and (1.4–416 μM) with a LOD of 3.0 nM. The sensor’s selectivity was evaluated using a 10-fold excess of interfering species such as malathion, parathion, carbofuran, ascorbic acid, and caffeic acid. Also, some metal ions (Ca^2+^, Mn^2+^, Fe^2+^, Cu^2+^, and ${SO}_{4}^{2-}$) 15-fold excess were studied. No significant change was observed in the presence of these interfering compounds, demonstrating that the surface was highly selective. The applicability of the sensor was investigated by DPV under an optimized condition using the standard addition method in real samples. The recovery rates of apple, cucumber, cabbage, and carrot were 94–95%, 95–97%, 94–95%, and 94–96%, respectively, and the relative standard deviation (RSD) was less than 5%.

**Figure 5:**
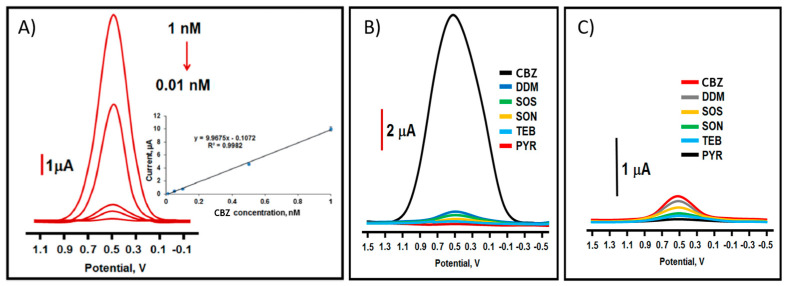
DPVs of (**A**) different concentrations of CBZ at MIP/CdMoO_4_/g-C_3_N_4_-15/GCE in PBS (pH 7.0), (**B**) interference study at MIP/CdMoO_4_/g-C_3_N_4_-15/GCE, and (**C**) interference study at NIP/CdMoO_4_/g-C_3_N_4_-15/GCE. Reproduced with permission from ref. [37].

Finally, Yola et al. [37] prepared a molecularly imprinted sensor based on CdMoO_4_/g-C_3_N_4_ nanocomposite to detect CBZ in fruit juice samples. First, the g-C_3_N_4_ was synthesized from melamine by a thermal condensation process at 600 °C over 90 min with a heating rate of 10.0 °C/min. Then, the CdMoO_4_/g-C_3_N_4_ nanocomposite was fabricated via one-pot hydrothermal synthesis (Figure 4C). This material was used to modify GCEs and to generate an imprinted MIP/CdMoO_4_/g-C_3_N_4_/GCE carbendazim sensor. The authors synthesized three different ratios of CdMoO_4_ to g-C_3_N_4_ (CdMoO_4_/g-C_3_N_4_–5/GCE, CdMoO_4_/g-C_3_N_4_–10/GCE, and CdMoO_4_/g-C_3_N_4_–15/GCE). The electrochemical performance of the nanohybrids was carried out in 1.0 mM [Fe(CN)_6_]^3−/4−^ by CV and EIS, determining that the most electrocatalytic material was CdMoO_4_/g-C_3_N_4_–15. Subsequently, the detection of CBZ by DPV was performed, determining a LOD of 2.5 pM with a linear range of 1 × 10^−3^–1 × 10^−5^ µM (Figure 5A), the lowest LOD value obtained with this carbon material. The authors state that incorporating CdMoO_4_ into g-C_3_N_4_ enhances the electrochemical activity of the sensor, improving the active surface area and the electron rate on the electrode surface. The recovery values of the sensor in apple and orange juice were close to 100%, demonstrating the sensor’s good accuracy. In terms of selectivity, the sensor demonstrated a strong affinity for carbendazim in the presence of other interfering substances such as diquat dibromide monohydrate (DMM), sodium sulfate (SOS), sodium nitrate (SON), tebuconazole (TEB), and pyraclostrobin (PYRA) (Figure 5B). For comparison, a non-imprinted polymer (NIP) was prepared on CdMoO_4_/g-C_3_N_4_–15/GCE. As shown in Figure 5C, the electrode did not provide satisfactory selectivity for determining CBZ.

### 3.4. Sensors Based on Other Carbon Structures

Different carbon structures such as carbon spherical shells (CSS) [38], N-doped carbon (NC) [39], carbon nanofibers (CNFs) [40,41,42], porous carbon derivatives [43,44], carbon nanospheres (CNS) [45], and carbon nanohorns (CNH)s [46] have been used to develop electrochemical sensors for CBZ detection. Part of these materials allow LOD values below 1.0 nM with applications in food, water, and biological samples.

**Figure 6:**
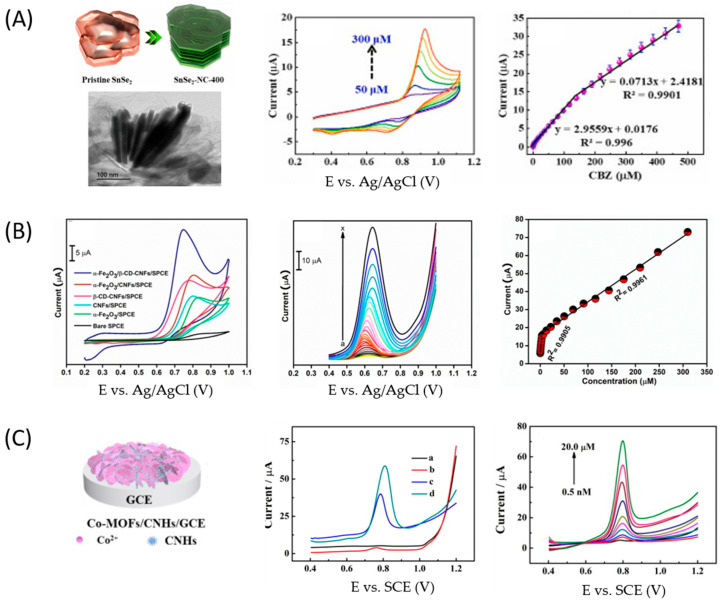
(**A**) Schematic illustration of the as-prepared pristine SnSe_2_ and SnSe_2_-NC structures; cyclic voltammograms of different concentrations of CBZ on SnSe_2_-NC-400/GCE at 50 mV s^−1^; calibration plot of CBZ; (**B**) CV curves of 100 μM CBZ at bare SPCE, α-${Fe}_{2}O_{3}$/SPCE, CNF/SPCE, β-CD-CNF/SPCE, α-${Fe}_{2}O_{3}$/CNF/SPCE, and α-${Fe}_{2}O_{3}$/β-CD-CNF/SPCE; DPV curves at α-${Fe}_{2}O_{3}$/β-CD-CNF/SPCE; Peak current responses vs. CBZ concentration; (**C**) schematic illustration for the Co-MOFs/CNHs/GCE; DPV curves of bare (a), Co-MOF/GCE (b), CNHs/GCE (c), and Co-MOFs/CNHs/GCE (d) in 0.1 M PBS (pH 6.0) containing 10 μM of CBZ; DPV of different concentrations of CBZ at Co-MOFs/CNHs/GCE in 0.1 M PBS (pH 6.0). Reproduced with permission from refs. [39,42,46].

Santhoshkumar et al. [39] prepared a SnSe_2_-grafted N-doped carbon composite (SnSe_2_-NC) through a hydrothermal method followed by different calcination temperatures, obtaining a 2D nanoflake structure (Figure 6A). This composite combines the conductive properties of the N-doped carbon material with the improvement in surface area due to SnSe_2_. A GCE was modified with the composites, and its electrocatalytic properties were evaluated by EIS and CV, where the material calcined at 400 °C (SnSe_2_-NC-400) was more suitable for electrochemical measurements due to its high electron transfer activity. The SnSe_2_-NC-400/GCE showed good electron transfer properties due to its high surface area and ionic conductivity. In the presence of CBZ, the sensor shows good accumulation behavior and excellent catalytic activity. Under optimal conditions, the amperometric response of CBZ shows a linear range between 0.002 and 139.38 µM and a LOD of 0.67 nM. Different interfering species, such as diuron, carbofuran, 4-nitrophenol, atrazine, diphenylamine, uric acid, ascorbic acid, Pb^2+^, Hg^2+^, and catechol, were evaluated. In all cases, no significant variation in current was observed, confirming the sensor’s suitability for real-sample analysis. Finally, the sensor was tested by monitoring CBZ in water and vegetable extracts by the standard addition method; unfortunately, the authors did not report recovery values.

In the case of Sebastian et al. [42], the electrode was modified with a novel dual-functional nanocomposite based on carbon nanofibers (CNFs), β-cyclodextrin (β-CD), and hematite (α-${Fe}_{2}O_{3}$) nanoparticles. The hybrid nanocomposite (α-${Fe}_{2}O_{3}$/β-CD-CNFs) was obtained through a sonochemical method and was used to modify screen-printed carbon electrodes (SPCEs) by the drop-casting method. The nanocomposite was characterized by SEM-EDS, XRD, FTIR, Raman spectroscopy, XPS, and EIS spectroscopy. When applied to the detection of 100 µM CBZ in 0.05 M PBS (pH 7.0), the weakest response was shown by the bare SPCE, indicating poor electrocatalytic activity of the electrode. Meanwhile, the best response was obtained by α-${Fe}_{2}O_{3}$/β-CD-CNF/SPCE with a 15 times higher current than SPCE, related to the superior electroactive surface of the modified SPCE. Once optimized, the sensor was used to detect CBZ by DPV at different concentrations, obtaining two linear ranges with a LOD of 1 nM under the linear range 0.06–309.75 µM (Figure 6B). The selectivity of the sensor was tested with different interferents (Mg^2+^, Cu^2+^, Zn^2+^, Cl^−^, ${NO}_{3}^{-}$, ${SO}_{4}^{2-}$, ${CO}_{3}^{2-}$, diphenylamine, thiabendazole, benomyl, chlorpyrifos, and diuron), where the maximum deviation was 4.3%, indicating good selectivity. Other parameters such as reproducibility (RSD 3.67%), repeatability (RSD 2.34%), and stability (reduction of 4.6% of current after 28 days) were also evaluated. Finally, the sensor detected CBZ in real samples prepared from orange, apple, tomato, and river water, where the recovery was between 96.6% and 102.2%.

Among the electrochemical sensors developed for CBZ detection with other carbon materials, the lowest LOD value was reached by Peng et al. [46]. The authors obtain a composite formed by the tremella-like co-metal organic framework (MOF) and carbon nanohorns (CNH) synthesized via a one-step electrochemical co-deposition technique. In this article, CNHs were decorated with Co-MOF and deposited onto GCE (Co-MOF/CNHs/GCE), combining the high surface area and active sites of Co-MOFs with the superior electrocatalytic properties and electronic conductivity of CNHs. The electrode was characterized by EIS, where the changes in charge transfer resistances (R_ct_) are noticeable. The R_ct_ value for GCE was 317.3 Ω, which increases to 573.4 Ω in the presence of Co-MOF. This result was expected because the Co-MOF blocked the diffusion of the redox mediator to the surface. The presence of CNHs in the electrode and Co-MOF (Co-MOF/CNHs/GCE) improved the electrode response compared to Co-MOF/GCE, decreasing the resistance to 136.7 Ω. These results were corroborated with the calculated electroactive specific surface areas (A), where the modified sensor showed a larger A (from 0.077 cm^2^ for GCE to 0.827 cm^2^ for Co-MOF/CNHs/GCE), providing more catalytic sites for CBZ. Following the oxidation of 10 µM CBZ in 0.1 M PBS (pH 6.0), the best response was obtained with Co-MOF/CNHs/GCE due to the synergistic effect between Co-MOF and CNHs. Under optimal analytical conditions (pH 6.0, 90 s of accumulation time, and 200 s of electrodeposition time for the preparation of Co-MOF/NHS), the CBZ calibration curve was constructed (Figure 6C). The sensor showed good linearity in the range of 0.5–20,000 nM, with a LOD value of 0.2 nM. The reproducibility (RSD 2.45%), repeatability (RSD 2.36%), and selectivity of Co-MOF/CNHs/GCE were evaluated with good results. Interferences such as K^+^, Na^+^, Cu^2+^, Cl^−^, ${NO}_{3}^{-}$, ${SO}_{4}^{2-}$, uric acid, ascorbic acid, fenitrothion, malathion, and thiabendazole were tested, and no influence was detected. Finally, the sensor detected CBZ in strawberry and cabbage samples. The recoveries varied from 97.3 to 101.6% (strawberry) and 99.0 to 103.5% (cabbage), demonstrating accuracy and reliability.

### 3.5. Sensors Based on Two Mixed Carbon Materials

Using two or more carbon-based materials is presented as an attractive alternative that enhances the characteristics of the sensor. In some cases, a synergy between both components is expected, improving the sensor’s sensitivity and increasing the electrocatalytic effect. Thus, it is common to find various combinations of materials that demonstrate the above in the literature.

Ilager et al. [47] prepared a nanohybrid mixing GO and g-C_3_N_4_. The authors state that the greater surface area of GO, accompanied by the chemical, thermal, electrical, and optical properties of GO and g-C_3_N_4_, could be more helpful in improving the electrode’s sensitivity. The electrode preparation includes modifying the GCE with GO/g-C_3_N_4_ by drop-casting and activating the modified electrode by running 20 cycles of cyclic voltammetry in a potential window of 0.4–1.4 V at 50 mV/s (PBS, pH 4.2). Compared to the bare GCE, the presence of the nanohybrid GO/g-C_3_N_4_ produces changes in the voltammetric behavior of CBZ, where a potential shift of 174 mV (from 1.106 to 0.932 V) and a current increase of 10-folds (from 3.39 to 30.01 μA) were observed, demonstrating the electrocatalytic effect of the nanohybrid. The quantification of CBZ was performed by square wave voltammetry (SWV), obtaining a linear range of 0.01–250 μM and a LOD value of 2.82 nM. They evaluated the influence of metal ions in the voltammetric response of CBZ (${CaCl}_{2},\;{CuSO}_{4},\;{FeSO}_{4},\;{KNO}_{3},\;{MnSO}_{4},\;{ZnCl}_{2},\;{MgCl}_{2},\;NaCl)$ and the sensor presented high selectivity. Finally, the sensor was applied to detect CBZ in different spiked water and soil samples, where the recovery was 91% and 98% for water and soil samples, respectively.

**Figure 7:**
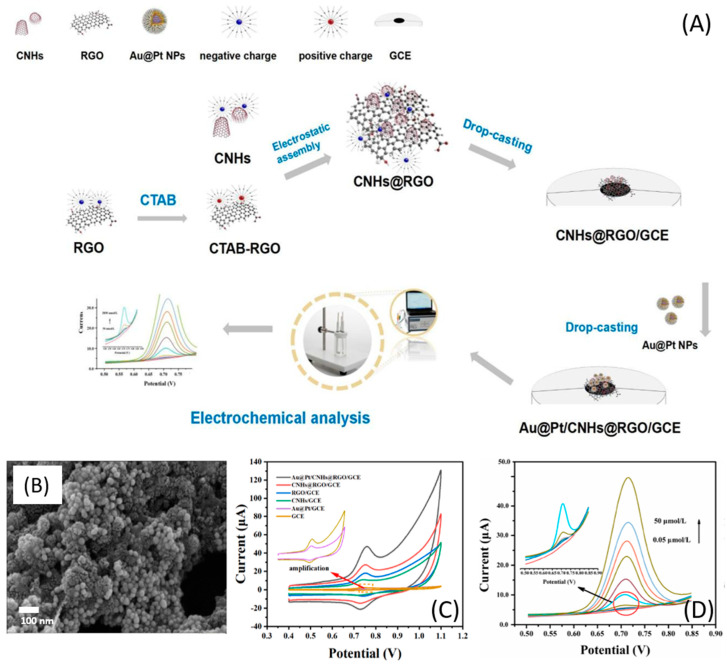
(**A**) Synthetic route of Au@Pt/CNHs@RGO/GCE and the sensing strategy for CBZ; (**B**) SEM image of Au@Pt/CNHs@RGO; (**C**) CV response of different electrodes with CBZ, and (**D**) DPV response of CBZ at different concentrations with Au@Pt/CNHs@RGO/GCE. Reproduced with permission from ref. [48].

Another alternative is a mixture of carbon-based materials with metallic nanoparticles. In this context, Li W. et al. [48] developed a sensor based on carbon nanohorns coated with reduced graphene oxide and gold-platinum core-shell nanoparticles (Au@Pt/CNHs@RGO/GCE). Both carbon-based materials could improve the sensitivity of the electrochemical detection of CBZ since both are rich in oxygen-containing functional groups and are able to form hydrogen bonds with the imino group in CBZ. Also, incorporating metallic nanoparticles can increase the electroactive surface and improve the electrocatalytic activity and homogeneity of the material. The experimental procedure was performed according to Figure 7A. The preparation of the modified GCE with CNHs@RGO was performed by drop-casting. Then, dispersion of both nanoparticles of Au@Pt was added, generating the electrode Au@Pt/CNHs@RGO/GCE. The SEM image (Figure 7B) showed that the Au@Pt NPs covered the surface of CNHs@RGO, expecting a synergistic interaction between materials, which was confirmed by the electrochemical characterization (Figure 7C). The cyclic voltammetric response of the electrodes in the presence of 50 μM CBZ confirmed that the catalytic effect of the mixture is better than that of the individual materials. Moreover, the carbon materials show high electron transfer ability and a large electroactive surface area, facilitating the incorporation of metal nanomaterials. The analytical parameters were obtained from DPV experiments (Figure 7D) following the response of CBZ at different concentrations. The linear range obtained was 0.05–50 μM with an LOD of 1.64 nM, the lowest LOD for electrodes modified with carbon-based mix materials. Regarding the interfering species, substances such as methyl, parathion, ${SO}_{4}^{2-}$, ${Cl}^{-}$, dichlorvos, ${Na}^{+}$, carbofuran, Zn^2+^, glucose, Cu^2+^, Pb^2+^, and urea had no significant effect on CBZ detection. However, diphenylamine, ascorbic acid, Cd^2+^, methamidophos, and especially diquat significantly interfered with the detection performance. Finally, the sensor was used for quantitatively detecting CBZ in carrot and orange juice with satisfactory recovery rates (between 90.3% and 117.9%) and RSD within 10.55%.

**Figure 8:**
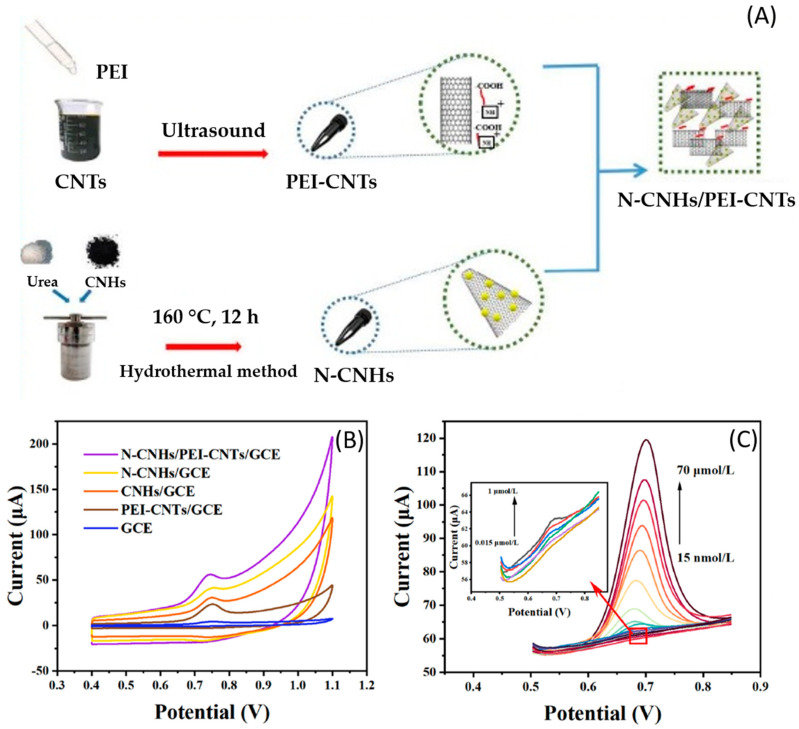
(**A**) Synthetic route of N-CNHs/PEI-CNTs; (**B**) CV response of different electrodes in CBZ, and (**C**) DPV response of CBZ at different concentrations with -CNHs/PEI-CNTs/GCE. Reproduced with permission from ref. [49].

Chen X. et al. [49] proposed a sensor based on nitrogen-doped carbon nanohorns (N-CNHs) and polyethyleneimine-modified carbon nanotubes (PEI-CNTs). N-CNHs increase the electrocatalytic activity and chemical reactivity of the surface, and PEI, a water-soluble polymer with a positive charge, makes the surface of CNTs have a positive charge, which could promote the electrostatic assembly of N-CNHs and PEI-CNTs. N-CNHs were obtained hydrothermally from CNHs, urea, and polytetrafluoroethylene at 160 °C for 12 h. PEI-CNTs were obtained in an ultrasound treatment lasting 240 min. Finally, both carbon-based materials were dispersed in DMF and treated with ultrasound for 2 h to get an N-CNHs/PEI-CNTs dispersion solution. Subsequently, CGE electrodes were modified by drop-casting (N-CNHs/PEI-CNTs/GCE). The scheme of synthesis and modification of electrodes is presented in Figure 8. The electrochemical detection of CBZ was by DPV at pH 7.0, obtaining a LOD of 4.0 nM and a linear range of 0.015–70 μM. The authors evaluated the sensor’s selectivity for 10 interferents (sodium chloride, zinc sulfate, methyl parathion, dichlorvos, carbofuran, diquat, urea, methamidophos, ascorbic acid, and cadmium chloride). It was determined that the presence of methamidophos, ascorbic acid, and Cd^2+^ produces interference in the detection of CBZ; therefore, the sensor is not highly selective. They evaluated the CBZ response in different water samples (tap, river, lake, and paddy water), obtaining a recovery between 87.3% and 117.7% with an RSD of 10.06%.

**Table 1:** Electrochemical sensors based on carbon materials for detecting carbendazim (2018–2023).

Carbon-Based Material	Working Electrode	Detection Method	Ep vs. Ag/AgCl 3M (V)	Lineal Range (µM)	LOD (nM)	Interfering Species	Sample Matrix	Ref.
Carbon Nanotubes	β-CD-MWCNT/BDDE	SWAdSV	1.23	0.67–11.2	200	Not described	River water	[50]
	Nd_2_Mo_3_O_9_/MWCNT/GCE	DPV	0.81	5 × 10^−5^–9.0	0.016	$K^{+},{Na}^{+},{NH}_{4}^{+},{Cu}^{2+},\;{{Cd}^{2+},Al}^{3+},{Cl}^{-},{CO}_{3}^{2-},$ ${PO}_{4}^{3-},{SO}_{4}^{2-}$, MP, FENI, MLT, DCP, BEN, TBZ, Thiophanate, TM, Fuberidazole, Glu, AA, vit B, vit C, vit E, DA, Serine	Paddy water	[20]
	CMC/MWCNTs/GCE	DPV	0.78	0.03–10	15	$K^{+},{Na}^{+},{Cl}^{-},{NO}_{3}^{-},$ Fru, Sucrose, vit C	Pear and kiwifruit	[51]
	MWCNT/Ca-ZnO/CPE	SWV	0.78	0.01–0.45	4.7	Not described	Tap and lake water, soil red, black, clay	[19]
	MoS_2_QDs@MWCNT/GCE	SWV	0.71	0.04–1.0	26	${Mg}^{2+},\;{Ca}^{2+},$$\;{Cl}^{-},$$\;{NO}_{3}^{-},$ AA, Carotene	Traditional Chinese medicine (*Platycodon grandiflorum*), pears	[52]
	MWCNT-COOH/GCE	SWV	0.81	0.30–20	60	MP, PQ, Niclosamide, inorganic substances	Cabbage, cucumber, potato	[53]
	MIP-MWCNT-CPE	DPV	0.79	0.1–100	31	BEN, Rabenzazole, TBZ	Surface water, groundwater, drinking water, seawater	[54]
	MWCNT/PtE	DP-ASV	1.09	0.25–2.5	61	Not described	Water, orange juice	[55]
	MoS_2_/CMC-MWCNT/GCE	DPV	0.73	0.04–100	7.4	Vit C, Vit B2, IMI, GLY, Endosulfan, Buprofezin, Fru, Sucrose, L-arginine, L-serine	Tea, rice	[18]
Graphene and its derivatives	GS/GCE	DPV	0.83	0.005–1.57	0.78	${Cu}^{2+},{Zn}^{2+},{Cd}^{2+},{Pb}^{2+},$ Hexachlorobenzene, Folpet, Triclosan, Picloram, HQ, phenol, BPA, Tetrabromobisphenol A, Parachlorophenol, p-aminophenol, PT, Dursban	Groundwater, soil, and cucumber	[21]
	ZnCdTe-rGO/CPE	DPV	0.88	0.099–11.8	92	AA, Citric acid	Orange juice	[56]
	NP-Cu/rGO/GCE	DPV	0.85	0.5–30	90	$K^{+},{Na}^{+},{Mg}^{2+},{Fe}^{2+},$${Cl}^{-},{CO}_{3}^{2-},$${NO}_{3}^{-},$${SO}_{4}^{2-},$ AMT, MP, FEN, TM	Pond water and lettuce	[57]
	Ti_3_C_2_T_x_/ERGO/GCE	DPV	0.78	0.002–10	0.67	$K^{+},{Na}^{+},{Mg}^{2+},{Cr}^{3+},$ ${Al}^{3+},{CO}_{3}^{2-},{NO}_{3}^{-},{PO}_{4}^{3-},$ MLT, MP, IMI, TCF, PROP	Cucumber and orange juice	[23]
	GdONRs/GA/GCE	DPV	0.80	0.01–75	3.0	DUR, CBF, DPA, UA, DA, CPZ, TRP, PYR, CPM	River water	[58]
	Ru-Asp-Arg-GQD/GCE	DPV	0.97	0.01–45	4.0	ACE, PROCh, CHI	Strawberry	[59]
	Gd_2_S_3_/NRGO/GCE	DPV	0.92	0.01–450	9.0	${Mg}^{2+},{Ca}^{2+},{CO}_{3}^{2-},$ DA, AA, UA, AMT, FEN, MP	River water	[60]
	GO/GCE	SWV	0.97	0.1–250	13.8	${Cu}^{2+},{Mg}^{2+},{Zn}^{2+},K^{+},\;{SO}_{4}^{2-},{NO}_{3}^{-},{Cl}^{-},{Na}^{+},$ ${Mn}^{2+}$, 2,4-D, DUR, amitrole, thymol, dichlorophen, linuron	Water and soils	[61]
	Sm_3_O_3_/RGO/GCE	DPV	1.03	0.019–0.198 0.29–421.3	3.0	$K^{+},{Hg}^{2+},{Pb}^{2+},$ MP, FENI, serotonin, HQ, CBF, DA	Orange juice, river water, vegetable extract, and lake water	[62]
	VMSF/BN-rGO/GCE	DPV	0.82	0.005–7.0	2.0	$K^{+},{Na}^{+},{Mg}^{2+},{PO}_{4}^{3-},$${CO}_{3}^{2-}$, starch, lignin, SDS, BSA, heme	Pond water and grape juice	[63]
	CoOx/ERGO/GCE	DPV	0.89	1×10^−5^–10	0.01	TM, MTX, ALA, DMT, CHL, PFF	Apple, tomato	[24]
Carbon Nitride	SnS_2_/g-C_3_N_4_/SPCE	DPV	0.64	0.02–0.9 1.4–416	3.0	${Mn}^{2+},\;{Ca}^{2+},\;{Fe}^{2+},\;{Cu}^{2+},$${SO}_{4}^{2-},$ MLT, PT, CBF, AA, CA	Apple, orange, cucumber, carrot	[36]
	MIP/CdMoO_4_/g-C_3_N_4_/GCE	DPV	0.50	1 × 10^−3^–1 × 10^−5^	0.0025	DMM, SOS, SON, TEB, PYR	Apple and Orange Juice	[37]
	Se-g-C_3_N_4_/SPCE	DPV	0.89	0.099–346.9	6.0	$K^{+},{Na}^{+},$4-AP, CAP, DPA, Glu, HQ, FENI, melatonin, BPA	Orange juice and vegetables	[1]
Other carbon structures	Gloves-Index Finger/CSS/SPCE	DPV	0.57 vs. Ag	0.1–1.0	47	DUR, PQ, FENI, Pyraclostrobin, TZ, Tecubonazole, Thiram	Apple, cabbage, orange juice	[38]
	CT-fC-Cu/SPCE	LSV	0.84	0.8–277	28	DUR, BENTA, CBF, DPA	Soil-washed water	[40]
	α-Fe_2_O_3_/β-CD-CNFs/SPCE	DPV	0.73	0.018–1.503 1.503–29.31	1.0	${Cu}^{2+},{Mg}^{2+},{Zn}^{2+},{SO}_{4}^{2-}$ ${NO}_{3}^{-},{Cl}^{-},{CO}_{3}^{2-}$, DPA, TBZ, BEN, CHL, DUR	Apple, orange, tomato, and river water	[42]
	SnSe_2_-NC/GCE	CV	0.89	0.002–139.38	0.67	${Hg}^{2+},{Pb}^{2+},$ DUR, CBF, 4-NP, AZ, DPA, UA, AA, CT	Water and vegetables	[39]
	MBC@CTS/GCE	DPV	0.85	0.1–20	20	$K^{+},{Ni}^{2+},{Ca}^{2+}{,{Mg}^{2+},}^{}$ ${Zn}^{2+},$ MP, IMI, PQ	Apple and tomato juice	[43]
	D-HPC/GCE	DPV	0.78	0.01–1.0	6.1	${Zn}^{2+},{Cl}^{-},{Ca}^{2+},{NO}_{3}^{-},$ ${Mg}^{2+}$$,\;{Cu}^{2+},$${SO}_{4}^{2-},$ Catechol, Glu, Metribuzin, Pymetrozine, DUR	River water, lettuce, and soil samples	[44]
	Gd_2_O_3_/f-CNS/GCE	DPV	0.99	0.5–552	9.0	${Na}^{+},$${Cl}^{-},$ MLT, FENI, FEN, CP, Glu, AA, UA	Blood serum, water, and vegetable samples	[45]
	Yb_2_O_3_/f-CNF/GCE	DPV	0.69	0.05–3035	6.0	$K^{+},{Ca}^{2+},{Mg}^{2+},{Fe}^{2+}$ ${Al}^{3+},{Cu}^{2+},{Cl}^{-},$${SO}_{4}^{2-},$ AMT, PT, TMX, CBF, 2,4,6-TCP, CAF, AA, CA	Carrot, radish, pond water, and lake water	[41]
	Co-MOFs/CNHs/GCE	DPV	0.83	5×10^−4^–20	0.2	$K^{+},{Na}^{+},{Cu}^{2+}$$,\;{Cl}^{-}$ ${NO}_{3}^{-},{SO}_{4}^{2-},$ UA, AA, FENI, MLT, TBZ	Strawberries and cabbage	[46]
Carbon-based material mix	GO/g-C_3_N_4_/GCE	SWV	0.93	0.01–250	2.82	${Zn}^{2+},{Cl}^{-},{Ca}^{2+},{NO}_{3}^{-},$ $K^{+},{Cu}^{2+}$ $,\;{SO}_{4}^{2-},{Mg}^{2+},$ ${Fe}^{2+}$	Water and soils	[47]
	β-CD/CNS@CNT/GCE	DPV	0.74	0.03–30	9.4	${Mg}^{2+},{NO}_{3}^{-},{Cl}^{-},{Ca}^{2+},$ ${Ni}^{2+},$$K^{+},{Br}^{-}$$,\;{Zn}^{2+},$ ${SO}_{4}^{2-},$ MP, FENI, IMI, PQ	Apple juice	[64]
	Au@Pt/CNH@RGO/GCE	DPV	0.74	0.05–50	1.64	${Cu}^{2+},{Cl}^{-},{SO}_{4}^{2-},{Zn}^{2+},$ ${Na}^{+},{Pb}^{2+},{Cd}^{2+},$ MP, Dichlorvos, CBF, Glu, DPA, AA, Diquat, Methamidophos,	Carrot and orange juice	[48]
	N-CNHs/PEI-CNTs/GCE	DPV	0.69	0.015–70	4.0	Not described	Water	[49]
	B/rGO-CPE	DPV	1.00	0.030–9.0	2.3	$K^{+},{Ca}^{2+},{Mg}^{2+},\;{Fe}^{2+}$ ${Al}^{3+},\;{Cu}^{2+},\;{Cl}^{-},\;{SO}_{4}^{2-},\;$ FEN, PQ, AMT, AA, CiA	Orange juice, lettuce leaves, drinking water, and wastewater	[65]

(a) Electrodes: β-CD: β-cyclodextrin; MWCNT: multi-walled carbon nanotubes; BDDE: boron-doped diamond electrode; GCE: glassy carbon electrode; CMC: carboxymethyl cellulose; Ca-ZnO: calcium-doped zinc oxide; CPE: carbon paste electrode; MoS_2_QD: MoS_2_ quantum dots; MIP: molecularly imprinted polymer; GS: graphene nanosheets; rGO: reduced graphene oxide; NP-Cu: nanoporous copper; ERGO: electrochemically reduced graphene oxide; GdONRs: Gadolinium oxide nanorods; GA: graphene aerogel; Ru-Asp-Arg-GQD: Ruthenium-aspartic acid-arginine graphene quantum dot; NRGO: N-doped reduced graphene oxide; GO: graphene oxide; VMSF: vertically-ordered mesoporous silica film; BN: Boron nitride; g-C_3_N_4_: graphitic carbon nitride; CSS: carbon spherical shells; CT-fC: Chitosan-carbon nanofiber; CNFs: carbon nanofibers; NC: N-doped carbon; MBC@CTS: Mung bean-derived porous carbon@chitosan composite; D-HPC: hierarchically porous carbon enriched with intrinsic defects; f-CNS: functionalized carbon nanosphere; CNHs: carbon nanohorns; CNS: carbon nanosheets; N-CNHs: nitrogen-doped carbon nanohorns; PEI: polyethyleneimine; B: biochar. (b) Interfering species:2,4,6-TCP: 2,4,6-trichlorophenol; 4-AP: 4-aminophenol; 4-NP: 4-nitrophenol; AA: ascorbic acid; ACE: acetamiprid; ALA: alachlor; AMT: ametrine; AZ: atrazine; BEN: benomyl; BENTA: bentazon; BPA: bisphenol A; BSA: bovine serum albumin; CA: caffeic acid; CAP: chloramphenicol; CBF: carbofuran; CHI: chlorothalonil; CHL: chlorpyrifos; CiA: citric acid; CPM: chlorpheniramine maleate; CP: chlorophene; CPZ: chlorpromazine; CT: catechol; DA: dopamine; DCP: dichlorophenol; DMM: diquat dibromide monohydrate; DMT: dimethoate; DPA: diphenylamine; DUR: diuron; FEN: fenamiphos; FENI: fenitrothion; Fru: fructose; Glu: glucose; GLY: glyphosate; HQ: hydroquinone; IMI: imidacloprid; MLT: malathion; MP: methyl parathion; MTX: metalaxyl; PQ: paraquat; PROCh: prochloraz; PROP: propham; PT: parathion; PYR: pyridoxine; PYRA: pyraclostrobin; RCF: trichlorfon; SDS: sodium dodecyl sulphate; SON: sodium nitrate; SOS: sodium sulfate; TBZ: thiabendazole; TEB: tebuconazole; TM: thiophanate-methyl; TMX: thiamethoxam; TRP: tryptophan; TZ: tartrazine; UA: uric acid.

## 4. Electrochemical Biosensors for CBZ Detection

Biosensors are analytical devices that contain two main components: a biological recognition element (enzymes, oligonucleotides, antibodies, aptamers, or cells) and a transducer (an electrode, in this case). Biosensors are noted for having high selectivity for the analyte attributed to the recognition element and the possibility of using different platforms and materials to immobilize or deposit the biological recognition element. Various configurations and types of biosensors can be found that achieve low detection limits and good analytical parameters with applications in multiple fields such as environmental monitoring, medical areas, and food. However, the main disadvantage is the instability and denaturation of the biological component, or in some biosensors, the need to work under physiological conditions; therefore, developing biosensors that meet all the necessities for rapid, selective, and low concentration detection is challenging [66,67]. 

Depending on the biosensor, the sensing principle changes and determines the detection signal for the analyte’s detection. When the bioelement is based on DNA, the electrode surface could immobilize single-strand (ss-) or double-strand (ds-) DNA, and the sensor could detect a hybridization process, the interaction of DNA with small molecules, or DNA damage. In all cases, the analyte can be detected through a label-free or label-based process. In a label-based process, the DNA sequence is modified with an organic dye, metal complexes, or enzymes, and the sensor’s response will be determined by the proximity of the molecule to the surface. Redox mediators such as methylene blue can be used [67]. In a label-free biosensor, the direct oxidation of DNA will determine the electrochemical response. When the bioelement is an aptamer, the biosensor is called an aptasensor. Aptamers are artificially short ss-DNA or synthetic RNA sequences that can specifically bind to target molecules by folding into distinct structures. Once the aptamer is immobilized on the substrate, the detection of the analyte can be performed, for example, by a direct assay using a redox probe such as [Fe(CN)_6_]^−3/−4^ [68]. After recognition, the conformational change of the aptamer blocks the access of the redox mediator to the surface of the electrode, and the detection of the analyte is observed by a decrease in current. Another bioelement commonly used for electrochemical biosensors is enzymes. Enzymes are biological molecules that catalyze chemical reactions, and they are composed of amino acids that generate proteins with 3D structures [69]. Enzymatic sensors can be divided into three types, depending on the result of the enzymatic reaction. In a first-generation enzymatic sensor, the measurement of the enzymatic product (e.g., H_2_O_2_) is detected on the electrode surface. A second-generation enzymatic sensor uses a redox mediator that is easily detected on the electrode. Finally, in a third-generation enzymatic sensor, the electron transfer between the enzyme and the electrode is directly measured without using a subproduct or a redox mediator [70].

Table 2 shows five carbon-based biosensors for CBZ detection. As can be seen, one of them used an enzyme as a bioelement, one used duplex DNA, and three articles used aptamers. Four electrodes used carbon-based materials (graphene nanoribbons, graphene aerogel, carbon nanohorns, and carbon nanotubes) in conjunction with gold nanoparticles to immobilize the biomolecule. Among the advantages of using carbon-based materials in developing biosensors, we highlight many sites for immobilization, a conductive surface, and the immobilization capacity of biomolecules using covalent and non-covalent methodologies [71]. In addition, using metal nanoparticles facilitates the load transfer process; it improves the homogeneity and quality of the hybrid material’s dispersion, and a synergistic response in the detection signal can be observed [72,73]. The recognition element is usually immobilized in the metal nanoparticle through a bond [74]. 

**Figure 9:**
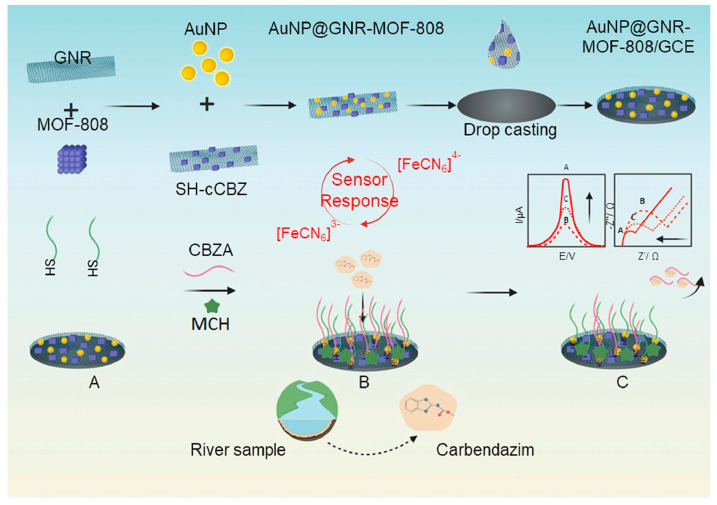
Scheme of aptasensor based on graphene nanoribbons MCH/CBZA/SH-cCBZ/AuNP@GNR/MOF/GCE. Reproduced with permission from ref. [75].

H. Khosropour et al., in 2022 [75], designed a dual-signal aptasensor based on gold nanoparticles/graphene nanoribbons (GNRs) and a Zr-based metal-organic framework (MOF-808) for carbendazim detection. GNRs are one-dimensional elongated graphene strips with a hexagonal lattice structure and can be synthesized by various methods [76]. In this work, GNRs were obtained by oxidative unzipping of MWCNTs. This carbon-based material has gained interest in developing sensors as it can decrease the overpotential and, in conjunction with metal oxides or metal nanoparticles to generate composites, can produce favorable electrochemical properties for voltammetric biosensing [77]. The experimental procedure for constructing the biosensor is presented in Figure 9. GNRs and MOF-808 were mixed during the synthesis of AuNPs to obtain AuNP@GNR/MOF-808; this composite was dispersed in DI water (1:1% *w*/*w*). With the dispersion, they modified GCE by drop-casting, then for 15 min incubated SH-complementary carbendazim aptamer (SH-cCBZ), followed by the addition of the sequence aptamer (CBZA) for 60 min at 37 °C, producing a hybridization between CBZA and SH-cCBZ. Finally, the electrode was coated with 6-mercapto-1-hexanol (MCH) to block active sites and avoid non-specific interactions. When the aptasensor recognizes the analyte, it separates the strands, interacting the CBZA sequence with CBZ. This results in an increase in current and a decrease in load transfer resistance in DPV and EIS measurements, respectively, in the presence of the redox mediator probe [Fe(CN)_6_]^3-/4^; thus, this aptasensor can detect CBZ by these two electrochemical techniques. The aptasensor presented a linear detection range of 1.0–100 fM y 0.8–100 fM. The detection limits determined by EIS and DPV were 0.4 and 0.2 fM, respectively, showing the lower detection limits for CBZ. The biosensor detected CBZ in tap water samples from Bangkok and the Chao Phraya Rivers. They determined good recoveries ranging from 97.5% to 102.8% and high selectivity for detecting CBZ in the presence of other pesticides as interferents.

**Figure 10:**
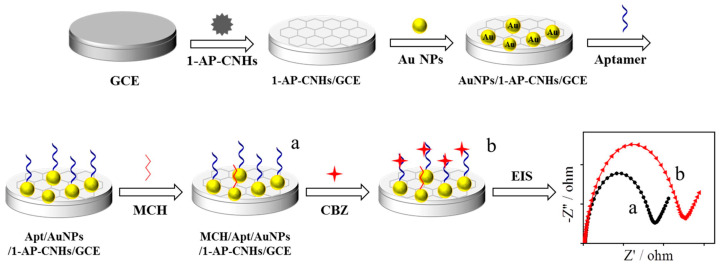
Schematic illustration of the aptasensor based on carbon nanohorns MCH/Apt/AuNPs/1-AP-CNHs/GCE. Reproduced with permission from ref. [78].

In 2019, C. Zhu et al. [78] developed an impedimetric aptasensor based on the modification of glassy carbon electrodes with a dispersion of carbon nanohorns (CNHs) and 1-aminopyrine (1-AP); the latter was used to improve the quality and homogeneity of the carbon-based material through p-p interactions between the pyrenyl groups of 1-AP and the nanohorn sidewalls [79]. CNHs have a conical structure with a sharp apical angle. Compared to carbon nanotubes, CNHs have higher surface defects, generating a good adsorption capacity, and may have superior electrolytic properties, making them suitable supports for constructing biosensors. Following the modification with the dispersion of 1-AP-CNHs, AuNPs with an average diameter of 16.7 nm were added. The thiolated aptamer sequence (SH-Apt) was immobilized, forming an Au-S bond on the electrode surface between SH-Apt and AuNP. Finally, the electrode surface was covered with MCH to avoid non-specific interactions. The aptasensor optimization was studied by cyclic voltammetry (CV) and electrochemical impedance spectroscopy (EIS) (Figure 10) by using a 10 µM AP concentration and an incubation time of 10 h when surface saturation was reached. The binding time of CBZ was 40 min. The detection limit of the developed CBZ aptasensor was 0.5 pg mL^−1^ with high selectivity for the analyte compared to interferers mixed with a 100-fold higher concentration of CBZ. After the detection of CBZ in orange juice and lettuce-enriched samples, the proposed method showed satisfactory recoveries between 95 and 102% in both cases, with RSDs less than 6.0%.

**Figure 11:**
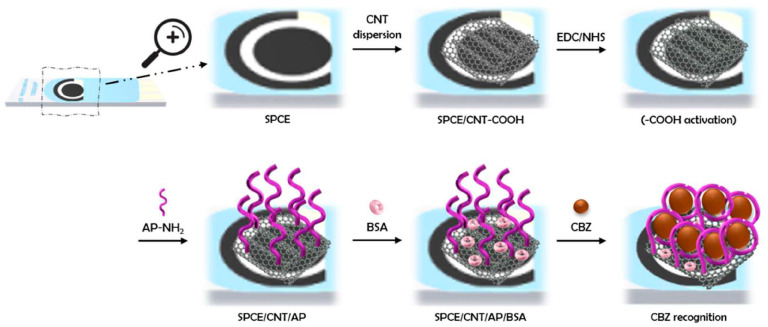
Schematic illustration of the aptasensor based on carbon nanotubes: SPCE/CNT/AP/BSA [80].

Finally, in 2023, Venegas et al. [80] reported a biosensor based on covalently immobilized aptamers on the surface of carbon screen-printed electrodes modified with oxidized multi-walled carbon nanotubes. The activation of the acid groups in carbon nanotubes was performed by the EDC/NHS reaction to immobilize an amino-terminal aptamer. Figure 11 shows each stage in the construction of the aptasensor. This article is the only publication where the bio-recognition element is immobilized covalently on the carbon nanomaterial’s surface without the need for metal nanoparticles, a simpler construction than those already reported. The aptasensor exhibited good CBZ selectivity, repeatability, and low LOD. It presented a recovery of 104.7% in a sample of CBZ-derived tomatoes.

**Table 2:** Electrochemical biosensors based on carbon materials for detecting carbendazim (2018–2023).

Carbon-Based Material	Bioelement	Working Electrode	Detection Signal	Technique	Lineal Range	LOD	Sample Matrix	Ref.
GNR	Aptamer	MCH/CBZA/SH-cCBZ/AuNP@GNR/MOF/GCE	[Fe(CN)_6_]^−3/−4^	DPV, EIS	(DPV) 0.8 fM–100 pM (EIS) 1.0 fM–100 pM	(DPV) 0.2 fM (EIS) 0.4 fM	Tap water, river water	[75]
GA	Duplex DNA	MF-Au-MGA/GCE	Methylene blue	DPV	1 × 10^−4^–10 pM	4.4 × 10^−5^ pM	Cucumber	[81]
CNHs	Aptamer	MCH/Apt/AuNPs/1-AP-CNHs/GCE	[Fe(CN)_6_]^−3/−4^	DPV	1–1000 pg/mL	0.5 pg/mL	Lettuce, orange juice	[78]
CNTs	Enzyme	HZnL-AuNPs/CNTs/CPE	pNP	SWV	10–100 μg/L	3.13 μg/L	Water samples	[82]
CNTs	Aptamer	BSA/AP-NH_2_/CNT/SPCE	[Fe(CN)_6_]^−3/−4^	DPV	0.19–10 ng/mL	0.83 ng/mL	Tomato	[80]

GNR: graphene nanoribbons; GA: graphene aerogel; CNHs: carbon nanohorns; CNTs: carbon nanotubes; MCH: 6-mercapto-1-hexanol; CBZA: carbendazim aptamer; SH-cCBZ: SH-complementary carbendazim aptamer; AuNP: gold nanoparticles; GNR: graphene nanoribbons; MOF: metal-organic framework; GCE: glassy carbon electrode; MF-Au: mulberry fruit-like gold nanocrystal; MGA: multiple graphene aerogel; Apt: aptamer; 1-AP-CNHs: 1-aminopyrene modified carbon nanohorns; CPE: carbon paste electrode; CNTs: carbon nanotubes; HZnL: lamellar zinc hydroxy nitrate; pNP: p-nitrophenol.

## 5. Conclusions and Perspectives

This review summarizes the latest carbon-based sensors and biosensors to detect carbendazim in complex matrixes. The carbon-based materials most commonly used included multi-walled carbon nanotubes, graphene and its derivatives, carbon nitride, carbon nanohorns, and carbon material mixes. Although the experimental conditions (pH, support electrolyte, electrochemical technique, etc.) are not the same for all the sensors, it is possible to find differences between the electrodes and the materials.

The electrochemical techniques used for the sensors presented in this review include cyclic voltammetry (CV), linear sweep voltammetry (LSV), square wave voltammetry (SWV), square wave adsorptive stripping voltammetry (SWAdSV), and differential pulse voltammetry (DPV). Of 37 articles revised, 78.4% used DPV, 13.5% used SWV, and the other techniques represented 2.7% each. Every sensor was capable of detecting the direct oxidation of CBZ. Since not all authors determine the electroactive area of the modified electrodes, it is difficult to establish a relationship between the electrode size or its electroactive area and the electrochemical response against CBZ. For the articles described in this review, the information on the diameters of each electrode is: glassy carbon electrode (GCE, d = 3 mm), screen-printed carbon electrode (SPCE, d = 4 mm), carbon paste electrode (CPE, d = 4 mm), platinum electrode (PtE, d = 0.5 mm), and boron-doped diamond electrodes (BDDE, d = 3 mm). Initial electroactive areas can change considerably once the surface is modified, depending on the material used, and may not be directly related to the surface area. Of the total articles included in this review, 69.1% used GCE, 14.3% SPCE, 11.9% CPE, and finally, BDDE and PtE, with 2.4% each. By analyzing the analytical performance of the sensors, the lowest LOD value reached was obtained using a modified GCE and DPV technique.

Among the carbon materials described, the sensors prepared with MWCNT and graphene’s derivatives had the best analytical parameters in terms of LOD values. When nanomaterials are combined, the sensors with the best response are those where carbon nanomaterials are mixed with metal nanoparticles instead of carbon-based materials. This could indicate that carbon-based materials do not necessarily have synergy when combined. In terms of electrocatalytic effect, the sensors modified with carbon nitride offer the lowest potentials for the electrooxidation of CBZ (Ep < 0.65 V) compared to the potentials observed when using CNTs and graphene (0.73 V < Ep < 1.23 V).

For electrochemical carbon-based biosensors, the detection of CBZ was indirect, i.e., they used redox mediators instead of the oxidation of CBZ. Aptasensors used the redox mediator [Fe(CN)6]^−3/−4^, one biosensor based on duplex DNA used Methylene Blue as a detection signal, and the enzymatic sensor used p-nitrophenol. Compared to sensors, biosensors allow for better selectivity and lower LOD values, mainly due to the biological element of recognition. As demonstrated, the biosensors that combined metal nanoparticles and carbon-based materials on the surfaces allowed lower detection limits and wider linear ranges. The enzymatic biosensor was the only one that used SWV as a detection method and presented the highest LOD value; meanwhile, the lowest LOD was obtained by an aptasensor using DPV as the technique. However, this result cannot be associated only with the detection method. Compared with other biosensors, enzymatic biosensors usually have higher LOD values than immunosensors or genosensors (sensors based on DNA), mainly due to the difficulty of adequately immobilizing the enzyme on the electrode surface.

Regarding the selectivity of the carbon-based (bio)sensors, it is still necessary to expand the studies to other interferers, such as benomyl, a pesticide with a similar chemical structure to carbendazim, which has been reported as the main interference in detection. Since only a few studies have tested the selectivity of the (bio)sensor against this interference agent, searching for new biosensors with simple designs that allow obtaining suitable analytical parameters is needed.

## Data Availability

Not applicable.
